# Law of Registration

**Published:** 1874-01

**Authors:** C. B. Nottingham


ATLANTA
MEDICAL AND SURGICAL JOURNAL.

Vol. XI.] JANUARY—1874. [No. 10.

### Original Communications.

### A LAW OF REGISTRATION OF BIRTHS, MARRIAGES AND DEATHS.

Whilst "too much legislation" is an evil that is deprecated by all thoughtful citizens, and although some unnecessary laws may already encumber our Book of Statutes, there are, in the judgment of many persons, some subjects underlying the great interests of the people of Georgia, that hitherto have been either totally neglected or only partially provided for by law, that have strong and daily increasing claims on the attention of legislators, and should not longer be overlooked, else evils far-reaching and irreparable will inevitably result. Of these subjects there is probably not one in regard to which appropriate legislation is of more pressing importance, or would be of more extensive and varied usefulness than that which constitutes the theme of this article.

For many years the want and indispensableness of a law providing for the registration of the Births, Marriages and Deaths among our people has engaged the thoughts of physicians, as well as other citizens, in different sections of the State. It was not known until last spring, at the annual meeting in the city of Atlanta, of the Georgia Medical Association, an organization that has on its roll of membership about six hundred names, embracing much of the intelligence and worth of the profession of the Commonwealth—that the thoughts took form and character, so far as physicians are concerned, in a determination to approach the Government formally and request the enactment of such a law. At that meeting—the largest in the four year's history of the Association—the matter was considered, and a resolution adopted under which a committee of seven members was appointed to perfect a bill with the assistance of the Attorney-General of the State, and to present said bill, together with a memorial setting forth its claims to consideration, to the Legislature at its session this winter.

Registry laws are not a new or untried thing. They have existed in the most civilized countries of Europe for a long period, and within the last few years they have been adopted in a number of the most advanced States of this Union. Wherever introduced they have effected great good; have been found practically useful in the advancement of science and the promotion of the public welfare, and people who have hitherto neglected them are now active and earnest in an effort to incorporate them among their statutes. Indeed, when considered in their high and sacred relations to property, family, and community, they must be adjudged to be of the highest dignity and of immense value.

In the absence of such provision in the Georgia Code the identification of individuals, the proof of the rightful ownership of property, and the evidence of important events in domestic life is even now, in the first half of the second century of our history as a people, sometimes a matter of diffiulty or even impossibility, and leads to the most serious embarrassments. If this has been found to be the case thus early, it is reasonable to suppose, nay, it can be predicted with unerring certainty that, as the State grows older and generations come and go, the difficulty will be of more frequent occurrence, the embarrassments will greatly multiply, and wrong and injustice, moral, social and pecuniary, will be sure to result in many instances and to many persons, unless provision is promptly made by the "law-making power" for the regular annual gathering and the placing permanently on record authentic and reliable information of the important events of birth, marriage and death; events that usually control the direction of estates, often involve the happiness of families, and to a large extent affect the interests and prosperity of the community. *This record*, full and complete, conclusive and satisfactory to all parties, can only be had under the provisions, requirements and authority of general official registration in the year in which the circumstances occur that are registered. Memory at best, is treacherous, and when the grave questions of lawful wedlock, of legitimacy, of geneological relations, of personal responsibilities and of title to property are in litigation, as is often the case in our courts of justice, or are matter of inquiry or dispute, as may not be a rare incident in that other high tribunal—one of the Star-Chambers of Society, something more than the oft-neglected and uncertain returns of the officiating clergyman in the matter of marriage, the only one of the three vitally important events in regard to which any legislation, whatever has been attempted, or the mere recollection of perhaps some aged individual in regard to either or all of them should be readily attainable in determining the weighty interests of heart and purse that may be in jeopardy.

But whilst the information that would be obtained on all the subjects embraced in the provisions of the law of registration now sought to be engrafted on the State Code would be valuable to us, in common with the great mass of citizens, in the fact that it would be eminently useful in subserving the ends of truth, justice and intelligence, and in the prevention of fraud, villainy and wrong; the *mortuary feature*—that relating to death and its cause especially, would be to us as medical men, the conservators of the public health and students of sanitary laws, of greatest interest and highest value. The truthful and reliable expositon furnished from year to year, in regard to the percentage of mortality of the aggregated and local populations; of the sanitary influence of the pursuits and habits of the people; of the death rate from preventable as well as from unavoidable disease; of the loss to the State of its true wealth in people from these causes; of the salubrity or insalubrity of the different sections of our territory; of the maladies most prevalent in particular localities; of the limits in which special affections are on the one hand most rife, or on the other, may be least likely to occur, or of districts where persons *suffering from or predisposed to* certain diseases would have the most propritious surroundings of cure or prevention in climate, air and water. The facts faithfully collected on these and kindred subjects as provided for in the death tables of a law of registration would be of incalculable value to us. When aggregated at the office of the Comptroller-General, they would constitute a map of life statistics that would open up to the profession of the State, material of the profoundest interest for investigation, analysis, comparison and deduction that would edify, instruct and illumine the medical mind, the rich fruits of which would be reaped by all classes of citizens throughout the entire State, in the improved capacities of their trusted advisers to ward off disease, effect cures and secure to them the greatest possible amount of health.

To a law of Registration of Births, Marriages and Deaths, there can be, as I conceive, under any circumstances, or from any point of view, but one solitary objection that is in any manner or in any degree whatever in the least rational, and that is the little expense attending its faithful execution. This objection, it is apprehended, can be met and obviated to a great extent by making the mchinery of the measure in both its theoretical and practical operation simple and uncomplicated. In our State no new office need of necessity be created, nor no new officer need be appointed, as it would be difficult to find more suitable agents to execute the law than the gentlemen known as the Tax Receivers of the several counties. Under the direction of the Comptroller General, these functionaries, when receiving tax-returns, if provided by that officer with the proper forms and nomenclature, could, and doubtless would, secure and return very full and complete information from their respective populations, and this probably, without any additional pay to that allowed for the service they now render as Receivers. If it is doubted, however, that they would be efficient in the discharge of the new-imposed duty without an increase of remuneration, then let them be allowed some extra compensation in the form of a minimum per capita fee to be paid out of the treasury of their counties, under the direction of the Grand Juries of the same. The amount thus paid would be so small that it would not be felt in the least burthensome by any class of tax-payers.

Can we not then, lay our Memorial for a Registry Law at the feet of the Legislators of the Commonwealth, with a reasonable hope of success? Can it be doubted that the judgment of the good policy of the enactment of the law pronounced last April by that large, patriotic and scientific body—the Georgia Medical Association, seconded as this judgment is by the voice of large numbers of other intelligent and influential citizens, will receive confirmation by those who are in place and authority? Will not the "law-makers," by appropriate legislation, at once place Georgia in the front rank of her sister States in the adoption of a measure that some of those States already have adopted and all soon will adopt, when the measure will cost comparatively nothing, is commended by the sanction of experience, challenges the homage of reason, lays tribute on the best feelings of the heart, and is certain to be serviceable to the present population, and of untold value to the coming generations of our descendants, her people?

Surely disappointment cannot be the result of the appeal we are directed to make to the Legislature at its approaching session. An enlightened judgment and far-seeing sagacity will control their action. Legislation will be had; the Bill will be enacted and suitable arrangements will be made for the thorough and faithful execution of the law.

C. B. Nottingham, M.D.

